# The Vital Roles of LINC00662 in Human Cancers

**DOI:** 10.3389/fcell.2021.711352

**Published:** 2021-07-20

**Authors:** Yuting He, Yating Xu, Xiao Yu, Zongzong Sun, Wenzhi Guo

**Affiliations:** ^1^Department of Hepatobiliary and Pancreatic Surgery, The First Affiliated Hospital of Zhengzhou University, Zhengzhou, China; ^2^Key Laboratory of Hepatobiliary and Pancreatic Surgery and Digestive Organ Transplantation of Henan Province, The First Affiliated Hospital of Zhengzhou University, Zhengzhou, China; ^3^Open and Key Laboratory of Hepatobiliary & Pancreatic Surgery and Digestive Organ, Transplantation at Henan Universities, Zhengzhou, China; ^4^Henan Key Laboratory of Digestive Organ Transplantation, Zhengzhou, China; ^5^Department of Obstetrics and Gynecology, The Third Affiliated Hospital of Zhengzhou University, Zhengzhou, China

**Keywords:** LINC00662, oncogene, proliferation, molecular mechanism, competing endogenous RNA

## Abstract

Long non-coding RNAs (lncRNAs) play crucial roles in many human diseases, particularly in tumorigenicity and progression. Although lncRNA research studies are increasing rapidly, our understanding of lncRNA mechanisms is still incomplete. The long intergenic non-protein coding RNA 662 (LINC00662) is a novel lncRNA, and accumulating evidence suggests that it is related to a variety of tumors in multiple systems, including the respiratory, reproductive, nervous, and digestive systems. LINC00662 has been shown to be upregulated in malignant tumors and has been confirmed to promote the development of malignant tumors. LINC00662 has also been reported to facilitate a variety of cellular events, such as tumor-cell proliferation, invasion, and migration, and its expression has been correlated to clinicopathological characteristics in patients with tumors. In terms of mechanisms, LINC00662 regulates gene expression by interacting with both proteins and with RNAs, so it may be a potential biomarker for cancer diagnosis, prognosis, and treatment. This article reviews the expression patterns, biological functions, and underlying molecular mechanisms of LINC00662 in tumors.

## Introduction

Cancer is a leading cause of death worldwide, second only to cardiovascular disease (Ferlay et al., 2018; Essa et al., 2020), and it represents a terrible threat to the health of mankind. In recent years, malignant-tumor incidence and mortality rates have increased in all countries at all income levels (Fidler et al., 2016; Torre et al., 2016). With the development of the Encyclopedia of DNA Elements project, most of the gene sequences in the genome have been clarified to be non-coding genome (Consortium, 2012; Consortium et al., 2020; Zhou et al., 2020). The non-coding RNAs (ncRNAs), which correspond to messenger RNAs (mRNAs), have traditionally been assumed to have many biological functions (Cech and Steitz, 2014; Matsui and Corey, 2017; Coker et al., 2019; Wang J. et al., 2019). In particular, current progress in sequencing technologies and large-scale genome sequencing projects have demonstrated that lncRNAs (>200 nucleotides) are crucial regulators of many human diseases, especially cancers (Xingming Jiang, 2015; Anastasiadou et al., 2018; Slack and Chinnaiyan, 2019).

Accumulating research has revealed that many lncRNAs play vital roles in cancer tumorigenicity (Yari et al., 2019; Feng et al., 2020) and progression (Zhu et al., 2017; Gong et al., 2020), are aberrantly expressed in a variety of cancers, and participate in many biological functions, such as cell proliferation (Ducoli et al., 2021; Shen et al., 2021), epithelial-to-mesenchymal transition (EMT) (Grelet et al., 2017; Zhao et al., 2017), invasion (Schmidt et al., 2016; Jin et al., 2019), migration (Liu L. et al., 2019; Zhao et al., 2018), angiogenesis (Zhao et al., 2020; Li et al., 2021), and differentiation (Luo et al., 2019; Tong et al., 2020). Moreover, lncRNAs can also function as competing endogenous RNAs (ceRNAs) (Wang et al., 2017; Zhou et al., 2019), transcription factors (Long et al., 2017; Ducoli et al., 2021), and in post-transcriptional processing (Zhang et al., 2018; Botti et al., 2019). All these findings suggest that lncRNAs may be able to serve as potential cancer biomarkers, ameliorating some of the current problems caused by cancer.

Several lines of evidence support an oncogenic role for LINC00662, the sequence for which is located on chromosome 19. Upregulation of LINC00662 expression has been detected in many cancers when compared to adjacent non-cancerous tissues. Research studies focusing on the oncogenic properties of LINC00662 have shown that it can be an oncogenic regulator in several tumor types in a variety of systems, including the respiratory, reproductive, nervous, and digestive systems.

In the present work, we have summarized the latest evidence concerning the abnormal expression of LINC00662, its associated clinical features, and molecular mechanisms, and we discuss both its prognostic and therapeutic values for malignant tumors.

## The Expression Levels, Biological Processes, and Clinical Characteristics of LINC00662 in Human Cancers

In recent years, largely due to the rapid development of high-throughput technologies, many cancer-related lncRNAs have received widespread attention (Li et al., 2017; Yu et al., 2017; Wang J. et al., 2018; Li Y. et al., 2020), including LINC00662. However, a possible pan-cancer role for LINC00662 has not been clear, so a comprehensive review of its expression levels, biological functions, and clinical features is warranted (see Table 1).

**TABLE 1:** Expression level and biological functions of LINC00662 in human cancers.

Human body systems	Cancer type	Expression	Property	Prognosis	Function	References
Respiratory system	lung cancer	up-regulated	oncogene	poor	proliferation and colony formation	Xu et al. (2020)
	lung cancer	up-regulated	oncogene	poor	invasion, migration and CSCs stemness	Gong et al. (2018)
	non-small cell lung cancer	up-regulated	oncogene	/	progression, proliferation, apoptosis, cell cycle arrest, migration and invasion	Lv et al. (2021)
Reproductive system	breast cancer	up-regulated	oncogene	poor	proliferation and migration	Cheng L. et al. (2020)
	breast cancer	up-regulated	oncogene	poor	/	Xiong et al. (2020)
	prostate cancer	up-regulated	oncogene	poor	tumorigenesis, proliferation, migration, invasion and apoptosis	Li et al. (2019)
	prostate cancer	up-regulated	oncogene	poor	proliferation, migration, invasion and apoptosis	Yao et al. (2020a)
	cervical cancer	up-regulated	oncogene	/	proliferation, metastasis, progression and the radioresistance	Wei et al. (2020)
Nervous system	glioma	up-regulated	oncogene	/	proliferation and migration	Geng et al. (2020)
	glioma	up-regulated	oncogene	poor	proliferation, and invasion	Wu et al. (2020)
	chordoma	up-regulated	oncogene	poor	malignant progression, proliferation, colony formation, invasion, migration, and glycolysis	Wang et al. (2020)
Digestive system	colorectal cancer	up-regulated	oncogene	poor	tumorigenesis, metastasis, cell cycle arrest at G2/M phase, proliferation, apoptosis, migration and invasion	Wang H. et al. (2019)
	colorectal cancer	up-regulated	oncogene	poor	proliferation, apoptosis, and invasion	Yao et al. (2020b)
	colon cancer	up-regulated	oncogene	poor	proliferation, migration and invasion, apoptosis	Cheng B. et al. (2020)
	hepatocellular carcinoma	up-regulated	oncogene	/	/	Tian et al. (2020)
	hepatocellular carcinoma	up-regulated	oncogene	poor	proliferation, migration, invasion and apoptosis	Guo et al. (2020)
	esophageal squamous cell carcinoma	up-regulated	oncogene	poor	proliferation, migration, invasion, viability and metastasis	Zhang et al. (2020)
	gastric cancer	up-regulated	oncogene	poor	proliferation, and chemo-sensitivity	Liu et al. (2018)
	oral squamous cell carcinoma	up-regulated	oncogene	poor	proliferation, apoptosis, migration and invasion	Xu et al. (2019)
	oral squamous cell carcinoma	up-regulated	oncogene	/	radioresistance	Chen et al. (2020)
Other systems	melanoma	up-regulated	oncogene	poor	proliferation, migration and invasion	Xia et al. (2020)
	acute myeloid leukemia	up-regulated	oncogene	/	malignant growth	Liu Y. et al. (2019)

### Respiratory-System Tumors

Globally, the leading cause of cancer deaths is lung cancer (LC) (Chen et al., 2016; Duma et al., 2019; Nasim et al., 2019). Numerous studies have confirmed that lncRNAs, including LINC00662, participate in LC progression (Yang et al., 2017; Wei et al., 2019; Pan et al., 2020; Zang et al., 2020). Xu et al. (2020) reported elevated LINC00662 expression in LC cells, which accelerated both their proliferation and colony formation. In addition, LINC00662 expression has been shown to promote invasion, metastases, and the stemness of LC stem cells as well (Gong et al., 2018). Moreover, Lv et al. (2021) reported that LINC00662 promoted the growth progression of non-small cell LC *in vivo* on the basis of enhanced proliferation in LC cells. LINC00662 may therefore be considered a promising diagnostic target for LC patients.

### Reproductive-System Tumors

For women, breast cancer (BC) is one of the most lethal cancers worldwide, and BC morbidity in China is gradually increasing (Fahad Ullah, 2019; Ding et al., 2020). The expressions of lncRNAs have been reported to be closely associated with BC (Kansara et al., 2020). Cheng L. et al. (2020) showed that LINC00662 expression was elevated in both BC tissues and cells when compared to normal BC cell lines and tissue. Furthermore, the silencing of LINC00662 was reported to significantly inhibit both BC cell proliferation and motility. In addition, Xiong et al. (2020) found that LINC00662 expression levels in BC patients showed a significant positive correlation with overall survival.

Although overall survival for patients with prostate cancer (PCa) is already high, PCa remains the most common tumor type in men (Li et al., 2019), and metastatic PCa remains incurable at present (Wang G. et al., 2018). Therefore, the identification of new therapeutic targets for PCa patients remains necessary. Interestingly, Li et al. (2019) demonstrated that LINC00662 was highly expressed both in PCa cells and in tissue samples compared to levels in para-cancerous tissues and a normal prostate epithelial cell line. While LINC00662 overexpression was shown to be positively correlated to both distant metastases and to shorter overall survival, *in vitro* LINC00662 silencing inhibited PCa cell proliferation and motility and promoted cell apoptosis. Yao et al. (2020a) also revealed that LINC00662 overexpression was positively associated with TNM staging, primary lesion size, lymph-node metastases, and distant metastases.

Cervical cancer (CC), a common malignant tumor in females, accounts for 529,800 newly diagnosed cases annually, with radio-resistance significantly reducing its therapeutic effect in many patients. Therefore, exploring the possible mechanisms governing CC tumorigenesis and progression remains important for the diagnosis and treatment of CC patients (Wei et al., 2020). In this regard, Wei et al. (2020) showed that LINC00662 expression was significantly upregulated both in CC tissues and in CC cells and that this high LINC00662 expression facilitated CC cell proliferation, motility, and radio-resistance. In contrast, knockdown of LINC00662 expression inhibited all of the above-mentioned CC cellular events. We therefore suggest that LINC00662 expression may be of therapeutic value as a diagnostic biomarker for reproductive-system tumors in clinical practice.

### Nervous-System Tumors

As the most lethal of all primary brain tumors, gliomas account for 80% of all central nervous system neoplasms (Geng et al., 2020). Accumulating evidence also indicates that the abnormal expression of lncRNAs participate in the occurrence and development of nervous-system tumors. For glioma patients, Wu et al. (2020) showed that LINC00662 overexpression was related to both unfavorable clinical characteristics and poor prognoses; however, the silencing of LINC00662 was shown to suppress glioma cell proliferation and invasiveness *in vitro*. Moreover, they also confirmed that knocking down LINC00662 expression in an *in vivo* nude-mouse model inhibited glioma growth.

Unlike gliomas, chordomas are uncommon malignant tumors and still merit the identification of novel biomarkers for diagnosis and treatment development. Both LINC00662 and RNF144B expressions have been shown to be aberrantly upregulated in chordoma tissues, and their knockdowns resulted in the attenuation of chordoma cell proliferation, colony formation, invasiveness, migration, EMT, and glycolysis (Wang et al., 2020).

Taken together, these studies suggest that LINC00662 serves as an oncogene in both glioma and chordoma and should be considered as a potential biomarker for treating these two tumor types that originate in the nervous system.

### Digestive-System Tumors

Colorectal cancer (CRC) is the deadliest and highest-incidence cancer worldwide, characterized by high rates of both metastasis and reoccurrence (Wang L. et al., 2019). Studies have reported that many lncRNAs participate in CRC progression, including MALAT1 (Xu et al., 2018) and GAS5 (Ni et al., 2019). Wang H. et al. (2019) found that LINC00662 was significantly upregulated in CRC cells and positively correlated with the degree of tumor differentiation, tumor stage, and lymphatic metastasis. The overexpression of LINC00662 was also an indicator of poor overall survival. However, Yao et al. (2020b) reported that the down-regulation of LINC00662 was also correlated with good patient prognosis and with the significant repression of CRC cell proliferation and promotion of apoptosis. The overexpression of LINC00662 was reported by Cheng B. et al. (2020) to enhance CRC cell invasiveness and migration and to dramatically accelerate CRC growth.

As the third most common cause of cancer-related deaths globally (He et al., 2020), hepatocellular carcinoma (HCC) represents an immense burden to society, with high rates of morbidity and mortality, particularly in China. Numerous research reports have shown that the expressions of lncRNAs (e.g., MIAT, HULC, and PDPK2P) are closely correlated with HCC tumorigenesis and progression. For LINC00662, Tian et al. (2020) reported that it was overexpressed in transforming growth factor beta-exposed HCC cells using existing transcriptomic data, and (Guo et al., 2020) reported that LINC00662 was up-regulated in HCC, positively correlated with patient survival, promoted HCC cell proliferation and motility, and reduced apoptosis.

Esophageal cancer is the eighth-most common cancer worldwide, with a 5-year survival rate lower than 20% (Hirano and Kato, 2019; Reichenbach et al., 2019). Histologically, esophageal cancer can be classified into squamous cell carcinoma and adenocarcinoma, as well as other types. Esophageal squamous cell carcinoma (ESCC) is especially prevalent in Linxian County, Henan Province, China. Zhang et al. (2020) demonstrated that the level of LINC00662 expression was upregulated in ESCC, and this was correlated with the adverse clinical characteristics for ESCC patients. Moreover, the knockdown of LINC00662 was shown to reduce ESCC cell proliferation, migration, and invasiveness.

The mortality rate of gastric cancer (GC) remains high due to its frequent diagnosis only at advanced stages, making it the third-most frequent cause of cancer-related deaths (Smyth et al., 2020). Liu et al. (2018) reported that high LINC00662 expression in GC tissues and cells was associated with poor patient prognoses compared to patients with low LINC00662 expressions. Furthermore, the knockdown of LINC00662 was shown to both suppress GC cell proliferation and to enhance GC chemo-sensitivity.

Among head and neck cancers, oral squamous cell carcinoma (OSCC) is considered highly aggressive and represents the top cause of deaths globally (Xu et al., 2019; Chen et al., 2020). Notably, Chen et al. (2020) revealed that compared to normal tissues, LINC00662 expression was elevated in OSCC and that blocking its expression ameliorated OSCC radio-resistance. Similarly, Xu et al. (2019) also demonstrated that the overexpression of LINC00662 was closely correlated with the adverse clinical characteristics of tumor size, tumor stage, and lymph node metastasis ascribed to its influences on OSCC cell proliferation and motility. The above studies shed new light on the value of lncRNAs for both therapeutic and prognostic purposes for digestive-system tumors and suggest that LINC00662 may be a promising biomarker for many digestive-system tumors.

### Tumors in Other Systems

In the United States, potentially fatal melanoma is the fifth-most prevalent cancer in males and the sixth-most prevalent cancer in females (Marco Rastrelli, 2014). Xia et al. (2020) showed that, in patients with melanoma, high levels of LINC00662 expression were associated with shorter survival times and that both melanoma tissues and cell lines showed high LINC00662 expression levels. In addition, the knockdown of LINC00662 was shown to restrain cell proliferation, migration, and invasiveness, and in *in vivo* experiments, LINC00662 expression was shown to facilitate tumor growth.

Acute myeloid leukemia (AML) is a malignant disease caused by myeloid hematopoietic progenitor cells. It is characterized by the aberrant proliferation of primitive and immature myeloid cells in both the bone marrow and peripheral blood (Ari Pelcovits, 2020). Even with the best current treatments, AML prognoses are poor, especially in patients 65 years or older (De Kouchkovsky and Abdul-Hay, 2016). Therefore, a better understanding of AML pathogenesis and its molecular mechanisms is necessary for the development of novel AML therapies. In AML, Liu Y. et al. (2019) demonstrated that LINC00662 was upregulated in AML cells and significantly promoted AML cell growth, while LINC00662 downregulation was reported to suppress AML cell growth and to accelerate apoptosis. Therefore, LINC00662 can be regarded as a promising prognostic and therapeutic target for patients with OSCC, melanoma, and AML.

## LINC00662 Mechanisms in Human Tumors

Studies have determined that lncRNAs are primarily localized to the nucleus and chromatin, indicating that their significant influence may be on DNA (West Jason et al., 2014; Sun et al., 2018). However, even though lncRNAs lack protein-coding ability, they also play important roles in a variety of cellular processes via many molecular mechanisms as described below. Some lncRNAs have *cis* epigenetic roles, interacting with proteins to mediate protein-coding gene expression (Wang et al., 2008), while other lncRNAs can interact with transcription factors to competitively suppress them, in a capacity known as a molecular decoy (Geisler and Coller, 2013). Some lncRNAs may also function as miRNA sponges to suppress miRNA activity (Cesana et al., 2011), modify enhancer activities, and modulate active-chromatin states (Wang et al., 2011). In addition, lncRNAs can also regulate gene expression post-transcriptionally by binding to antisense mRNAs (Carrieri et al., 2012). In terms of the molecular mechanisms by which LINC00662 acts in cancers, studies have increasingly shown that LINC00662 mainly participates by regulating target miRNA by acting as a ceRNA (Shuai et al., 2020).

### LINC00662 Can Act as a ceRNA in Post-transcriptional Regulation

**FIGURE 1:**
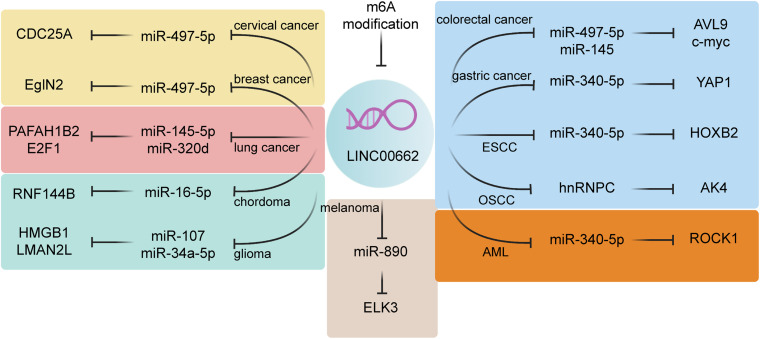
The ceRNA network of LINC00662. LINC00662 is involved in a variety of tumors through the sponging of miRNAs, including miR-497-5p, miR-145-5p, miR-320d, miR-16-5p, miR-16-5p, miR-107, miR-34a-5p, miR-340-5p, hnRNPC, and miR-890. Yellow represents reproductive-system tumors; green represents nervous-system cancers, and blue represents digestive-system tumors. The level of LINC00662 expression is regulated by m6A-level modifications.

As long as RNA transcripts (e.g., lncRNAs, circular RNAs, and mRNAs) can combine with miRNA response elements (MREs), they are considered to have the ability to act as ceRNAs (Karreth and Pandolfi, 2013; Li X. et al., 2020). The examples mentioned above are entirely ncRNAs, so the ceRNA proposition suggests that miRNAs provide the link between ncRNAs and protein-coding RNAs. This idea of lncRNA/miRNA/mRNA network has previously been advocated as having an indispensable role in both tumorigenesis and the development of many human tumors. Many studies have confirmed that LINC00662 is an important lncRNA member and participates in this lncRNA/miRNA/mRNA network. The ceRNA functions attributed to LINC00662 are presented in Figure 1 and Table 2.

**TABLE 2:** CeRNA networks related to LINC00662 in multiple cancers.

CeRNA function of LINC00662	Cancer type	References
miR-145-5p/PAFAH1B2 axis	lung cancer	Xu et al. (2020)
miR-320d/E2F1 axis	non-small cell lung cancer	Lv et al. (2021)
miR-497-5p/EglN2 axis	breast cancer	Cheng L. et al. (2020)
miR-497-5p/CDC25A axis	cervical cancer	Wei et al. (2020)
miR-34a-5p/LMAN2L axis	glioma	Geng et al. (2020)
miR-107/HMGB1 axis	glioma	Wu et al. (2020)
miR-16-5p/RNF144B axis	chordoma	Wang et al. (2020)
miR-497-5p/AVL9 axis	colorectal cancer	Wang H. et al. (2019)
miR-145/c-myc axis	colorectal cancer	Yao et al. (2020b)
miR-340-5p/HOXB2 axis	esophageal squamous cell carcinoma	Zhang et al. (2020)
miR-497-5p/YAP1 axis	gastric cancer	Liu et al. (2018)
hnRNPC/AK4 axis	oral squamous cell carcinoma	Chen et al. (2020)
miR-890/ELK3 axis	melanoma	Xia et al. (2020)
miR-340-5p/ROCK1 axis	acute myeloid leukemia	Liu Y. et al. (2019)

### LINC00662/miR-340-5p/mRNA for ROCK1/HOXB2

Liu Y. et al. (2019) demonstrated that LINC00662 could mediate miRNA-mRNA crosstalk interactions in AML by showing that LINC00662 combined directly with miR-340-5p and decreased its expression level. When LINC00662 was silenced, miR-340-5p levels were then significantly upregulated, AML cell proliferation was suppressed, and cell apoptosis increased. The restraint of miR-340-5p clearly attenuated the inhibitory effect of LINC00662 on proliferation suppression. In addition, decreased Rho-associated protein kinase 1 (ROCK1) expression was also shown to reverse the inhibitory effects of LINC00662 and miR-340-5p on proliferation. We therefore consider ROCK1 to be the target of miR-340-5p in AML cells. Currently, another important ESCC study has also reported a different ceRNA network related to the miRNA/mRNA axis. Zhang et al. (2020) showed that LINC00662 functioned as a ceRNA to sponge miR-340-5p and miR-340-5p led directly to the downregulation of homeobox B2 (HOXB2). Therefore, LINC00662 can upregulate HOXB2 expression and promote the development and progression of ESCC. In contrast, both the inhibition of HOXB2 and the upregulation of miR-340-5p slowed ESCC tumorigenesis. Taken together, these studies emphasize the vital roles of ceRNA interactions in mediating biological processes in both AML and ESCC.

### LINC00662/miR-497-5p/mRNA EGLN2/CDC25A/AVL9/YAP1

Mechanistically, Cheng L. et al. (2020) found that LINC00662 promoted BC cell proliferation via the regulation of the miRNA/mRNA axis. Their bioinformatics analysis revealed that LINC00662 could be a sponge for miR-497-5p, and further BC studies showed that LINC00662 directly combined with it resulting in its downregulation. In addition, LINC00662 was shown to directly target Egl-9 family hypoxia inducible factor 2 (EGLN2), which was also considered to be a direct target for miR-497-5p. Therefore, this study revealed that LINC00662 overexpression accelerated BC cell growth via competitive binding with miR-497-5p and promoting EglN2 expression. Mechanistically in CC, LINC00662 was shown to also interact competitively with miR-497-5p, similar to its interaction in BC cells. In this study, Wei et al. (2020) showed that LINC00662, as a ceRNA, promoted CC progression and radio-resistance via the sponging of miR-497-5p, resulting in increased CDC25A levels. Therefore, this lncRNA/miRNA/mRNA axis in CC accelerated the malignant behavior of CC cells and the targeting of LINC00662 may enhance radio-resistance in these cells. In a CRC study, LINC00662 was found to exert a ceRNA function via the binding/decrease of miR-497-5p, leading to the overexpression of AVL9 (Wang H. et al., 2019). Collectively, LINC00662 appears to play a crucial role in development of CRC by modulating the miR-497-5p/AVL9 axis, shedding new light on CRC pathogenesis. Moreover, one of the vital regulatory mechanisms related to GC was the LINC00662/miR-497-5p/YAP1 ceRNA network proposed by Liu et al. (2018), where the knockdown of LINC00662 in GC cells was shown to suppress yes-associated protein 1 (YAP1) expression via the sponging of miR-497-5p. Furthermore, YAP1 has been shown to be a crucial downstream regulator of Hippo signaling and a mediator of GC-cell proliferation. Therefore, LINC00662 represents a potentially new biomarker for patients with BC, CC, CRC, and GC.

### LINC00662/miR-16-5p/mRNA for RNF144B

Both LINC00662 and RNF144B have been shown to be aberrantly upregulated in chordoma tissues, and the knockdown of either LINC00662 or RNF144B impeded chordoma cell proliferation, colony formation, invasiveness, migration, EMT, and glycolysis. In addition, RNF144B overexpression was reported to reverse the effects of LINC00662 knockdown. Additionally, miR-16-5p was shown to target RNF144B and be a potential target for LINC00662. Thus, the relationship between LINC00662 and RNF144B was established through demonstrating their miR-16-5p interactions, and further studies also showed that the stable knockdown of LINC00662 inhibited tumor growth *in vivo* (Wang et al., 2020). These findings suggest that LINC00662 may also be an important therapeutic target for chordoma clinically.

### LINC00662/miR-107/mRNA for HMGB1

Wu et al. (2020) showed that LINC00662 mechanistically regulated the miR-107/HMGB1 axis as a ceRNA. Moreover, the upregulation of high-mobility group box 1 protein (HMGB1) was reported to weaken the inhibition of glioma growth caused by the knockdown of LINC00662, suggesting that HMGB1 should be considered an miR-107 target. As a DNA-binding protein, HMGB1 is well-conserved, and many studies have shown that it accelerates the development of gliomas.

### LINC00662/miR-34a-5p/mRNA for LMAN2L

Geng et al. (2020) reported that LINC00662 also acted as a ceRNA through miR-34a-5p binding and the resulting upregulation of lectin, mannose-binding 2-like protein (LMAN2L). For measures of functionality, the downregulation of LINC00662 has been shown to significantly suppress both cell proliferation and clonogenicity. Interestingly, inhibitors of miR-34a-5p (including LINC00662) have been reported to partially weaken the inhibitory effect on cell proliferation and migration induced by LINC00662 silencing in glioma cells. Therefore, these results suggest that the LINC00662/miR-34a-5p/LMAN2L axis can modulate glioma progression, bringing a new perspective to both glioma diagnosis and possible therapeutic methods for glioma patients.

### LINC00662/miR-145-5p/mRNA for PAFAH1B2

Mechanistically, Xu et al. (2020) further revealed that LINC00662 functions as an LC oncogene (acting as a ceRNA) through the binding and downregulation of miR-145-5p. In addition, the knockdown of LINC00662 was shown to elevate miR-145-5p levels and result in the downregulation of platelet activating factor acetylhydrolase 1B catalytic subunit 2 (PAFAH1B2). Specifically, Xu et al. (2020) also found that miR-145-5p could combine with the 3′untranslated region (UTR) of PAFAH1B2 for negative regulation and suppressing miR-145-5p blocked the LC-repressive effect of LINC00662 silencing.

### LINC00662/miR-890/mRNA for ELK3

LINC00662 has been reported to combine with miR-890, with ELK3 being the downstream gene target of miR-890. Furthermore, miR-890 has also been shown to negatively regulate ELK3 expression. Using rescue assays, the overexpression of ELK3 was shown to reverse the inhibitory effects of either LINC00662 knockdown or miR-890 mimics on the cell proliferative, migratory, and invasive abilities of melanoma (Xia et al., 2020).

### LINC00662/miR-320d/mRNA for E2F1

LINC00662 has also been shown to act as an miR-320d sponge in non-small cell LC cells, and E2F transcription factor 1 (E2F1) is known to be a target for miR-320d in these cells. In addition, exosomal LINC00662 was reported to accelerate non-small cell LC progression via the sponging of miR-320d *in vitro*, and this exosomal LINC00662 also significantly enhanced non-small cell LC growth. Thus, this exosomal LINC00662 study showed that non-small cell LC progression was promoted through modulation of the miR-320d/E2F1 axis and expands our understanding of potential exosomal LINC00662 mechanisms. LINC00662, miR-320d, and E2F1 may therefore all serve as potential targets for non-small cell LC therapies (Lv et al., 2021).

### LINC00662/hnRNPC/mRNA for AK4

Chen et al. (2020) reported that LINC00662 could interact with both heterogeneous nuclear ribonucleoprotein C (hnRNPC) and adenylate kinase 4 (AK4) in OSCC and that LINC00662 mainly interacted with hnRNPC protein to regulate AK4 mRNA stability, resulting in AK4 protein overexpression. Interestingly, LINC00662 was shown not to be able to interact with hnRNPC directly, and its modulation of OSCC cell radiosensitivity was shown to be through hnRNPC-modulated AK4. In fact, the knockdown of LINC00662 actually enhanced OSCC-cell radiosensitivity via the upregulation of AK4.

### LINC00662/miR-145/mRNA for c-myc

The results of a bioinformatics analysis reported miR-145 to be a speculative miRNA target for LINC00662. In addition, LINC00662 was shown to directly interact with miR-145 and to reduce its expression in CRC. The upregulation of miR-145 was also reported to attenuate CRC cell growth and to accelerate apoptosis, while its suppression was shown to markedly reverse the inhibitory effect of LINC00662 knockdown on CRC cell growth. In addition, the recovery of c-myc expression was reported to partially reverse the inhibitory effects on CRC cell growth mediated either by LINC00662 low-expression or by miR-145 overexpression. Taken together, this study indicates that LINC00662 modulated CRC cell biology by raising c-myc levels via its binding with miR-145 and that the LINC00662/miR-145/c-myc axis plays a crucial role in regulating CRC cell growth (Yao et al., 2020b).

### The Interaction Between LINC00662 and Protein

In addition to acting as a ceRNA, LINC00662 has also been shown to influence genomic methylation through its interactions not only with miRNA but also with protein (Guo et al., 2020). This research demonstrated that the effect of LINC00662 was mainly on the expressions of methionine adenosyltransferase 1A (MAT1A) and S-adenosylhomocysteine hydrolase (AHCY); key enzymes for the production of S-adenosylmethionine (SAM) and S-adenosylhomocysteine (SAH). On the one hand, as previously described, LINC00662 exerted its ceRNA function via the MAT1A 3′UTR by direct sponging, thereby reducing MAT1A mRNA and downregulating MAT1A protein expression. On the other hand, and more importantly, LINC00662 was also shown to directly interact with AHCY protein, thereby facilitating AHCY instability via the upregulation of its ubiquitin-mediated degradation (Guo et al., 2020). Taken together, LINC00662 has also been shown to participate in the activation of multiple oncogenes through protein interactions, shedding additional light on possible LINC00662 mechanisms.

## Pathway Related to LINC00662 Function in Cancers

**FIGURE 2:**
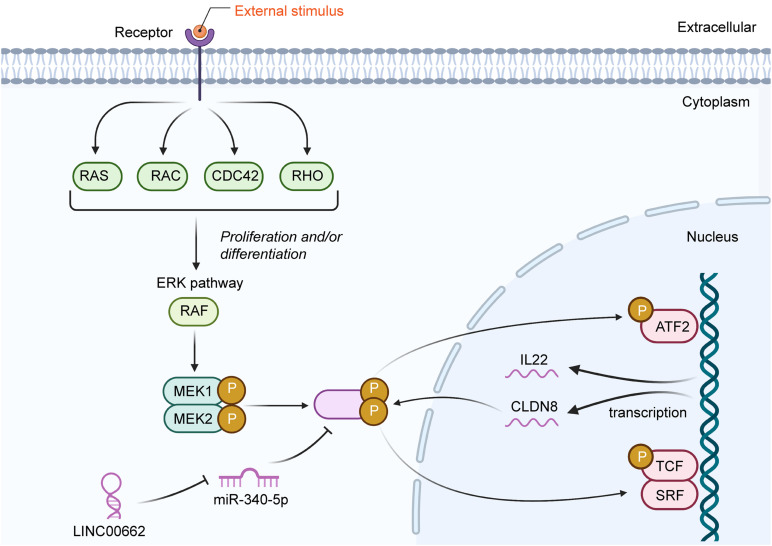
A schematic diagram of LINC00662 participation in the ERK signaling pathway. LINC00662 promotes p-ERK via binding to (and suppressing) miR-340-5p. Both CLDN8 and IL22 are target genes of ERK, and CLDN8 promotes p-ERK simultaneously.

**FIGURE 3:**
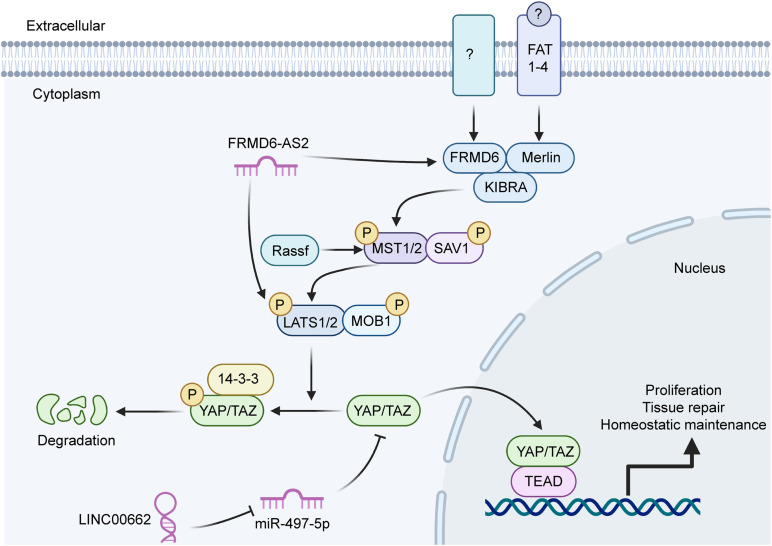
A schematic representation of LINC00662 involved in the Hippo signaling pathway. LINC00662 regulates YAP1 through the sponging of miR-497-5p, similar to the ERK signaling-pathway mechanism. Activation of the Hippo signaling pathway promotes cell proliferation, tissue repair, and homeostatic maintenance.

A variety of signaling pathways are active in cancer cells and are indispensable for their cellular processes. Recent evidence has indicated that lncRNAs, including LINC00662, are involved in many of these signaling pathways (Table 3). The extracellular signal-related kinase (ERK) signaling pathway is a vital contributor to many cellular processes and to the survival of cancer cells (Wen et al., 2019). The ERK family has five main members (ERK1–5); ERK1 and ERK2 are involved in regulating many biological processes, including meiosis and mitosis (Hu et al., 2019). LINC00662 has been shown to activate ERK signaling by raising the expression levels of both claudin 8 (CLDN8) and interleukin 22 (IL22) via the targeting of miR-340-5p (Cheng B. et al., 2020). These two genes play important roles in the pathogenesis of several intestinal diseases, including colon cancer, by promoting cell growth and metastasis (Figure 2). The Hippo signaling pathway has also become recognized as being important in GC. Liu et al. (2018) previously found that this pathway was linked to many cellular events via many biological molecules. Specifically, a decrease in LINC00662 expression has been shown to significantly reduce YAP1 expression, resulting in modulation of YAP1-mediated GC cell proliferation by the sponging of miR-497-5p (Figure 3). In addition, the Wnt/β-catenin signaling pathway also is of great importance for regulating cell proliferation in tumors. Xu et al. (2019) reported that the expression of LINC00662 promoted the activation of the Wnt/β-catenin signaling pathway. In their study, the upregulation of LINC00662 significantly increased Wnt3a and β-catenin proteins, while the knockdown of LINC00662 inhibited these proteins and blocked the overall activation of the Wnt/β-catenin signaling pathway. These studies indicate that LINC00662 may exert its oncogenic functions by regulating mitogen-activated protein kinase (MAPK)/ERK signaling, Hippo signaling, and the Wnt/β-catenin signaling pathway.

**TABLE 3:** Signaling pathways related to LINC00662.

Pathways related to LINC00662	Cancer type	PMID
MAPK/ERK signaling pathway	colon cancer	Cheng B. et al. (2020)
Hippo signaling pathway	gastric cancer	Liu et al. (2018)
Wnt/β-catenin signaling pathway	oral squamous cell carcinoma	Xu et al. (2019)

## Potential Clinical Application of LINC00662

### As a Biomarker for Cancer Diagnosis

In recent years, accumulating evidence has indicated that lncRNAs could be detected in plasma and tissues as biomarkers for the diagnosis of cancers (Li et al., 2019). Interestingly, LINC00662 has been found to have a potential diagnostic value for patients with lung cancer (Gong et al., 2018), chordoma (Wang et al., 2020), colorectal cancer (Wang H. et al., 2019; Yao et al., 2020b), gastric cancer (Liu et al., 2018), and acute myelocytic leukemia (Liu Y. et al., 2019). Gong et al. (2018) observed that the LINC00662 expression level in lung cancer tissues was significantly higher than that in adjacent normal tissues. LINC00662 expression was also upregulated in colorectal cancer tissues, betokening potential application of LINC00662 in colorectal cancer diagnosis (Wang H. et al., 2019; Yao et al., 2020b).

### As a Prognostic Biomarker for Cancer

Based on the recent studies, the overexpression of LINC00662 was significantly linked to poor prognosis in different cancer types. Guo et al. (2020) demonstrated that the increased expression of LINC00662 was associated with lower overall survival (*P* = 0.0071) and recurrence-free survival rate (*P* = 0.0064) in HCC. Gong et al. (2018) found that LINC00662 expression level in lung cancer was higher in advanced TNM stages. Therefore, overexpression of LINC00662 led to poor prognosis of lung cancer. Similarly, cervical cancer patients with high LINC00662 expression level had shorter overall survival time and relapse free survival time (Wei et al., 2020). Xu et al. (2019) found that in OSCC, LINC00662 expression was significantly correlated with tumor size, TNM stage, and lymph node metastasis. Many researches had reported that LINC00662 was a prognostic biomarker for patients with prostate cancer (Li et al., 2019), lung cancer (Wang et al., 2020), glioma (Geng et al., 2020; Wu et al., 2020), melanoma (Xia et al., 2020), colon cancer (Cheng B. et al., 2020), colorectal cancer (Wang H. et al., 2019), and gastric cancer (Liu et al., 2018).

### As a Therapeutic Target for Cancer

Though not much study has been done on the therapeutic value of LINC00662 for cancer patients, studies on LINC00662 in tumor have provided the probability of LINC00662 as a therapeutic target for cancers. Xia et al. (2020) found that suppression of LINC00662 repressed cell proliferation, migration, and invasion of melanoma, which indicated that LINC00662 could be a promising therapeutic target for patients with melanoma. Xu et al. (2019) discovered that downregulation of LINC00662 inhibited the proliferation, migration, and invasion abilities of OSCC cell, and promoted apoptosis. In *vivo*, LINC00662-depleted U87 cells were injected into the BALB/C immunodeficiency mice, and the tumor volumes and weights of the LINC00662 knockdown group were significantly decreased. Therefore, silenced LINC00662 suppressed glioma proliferation *in vivo*, proving that LINC00662 could serve as therapeutic potential in glioma (Geng et al., 2020). Moreover, LINC00662 also could be a novel therapeutic target for treatment of chordoma (Wang et al., 2020), prostate cancer (Li et al., 2019), breast cancer (Cheng L. et al., 2020), lung cancer (Gong et al., 2018), AML (Liu Y. et al., 2019), gastric cancer (Liu et al., 2018), and colorectal cancer (Wang H. et al., 2019; Yao et al., 2020b).

## Conclusion

Technological developments have provided efficient research tools for exploring lncRNAs and have led to the discovery of many lncRNAs in recent years (Ma et al., 2018; Xing et al., 2021). Accumulating research has identified crucial roles for lncRNAs in both tumor occurrence and progression (Peng et al., 2017), and the dysregulation of LINC00662 is among those identified as leading to a variety of cancers. The transcription site for LINC00662 is located on the 19th chromosome from site 28,281,401 to 28,284,848. Consistently, LINC00662 has been shown to be overexpressed in many different tumors, including lung cancer, breast cancer, cervical cancer, prostate cancer, chordoma, glioma, gastric cancer, and hepatocellular carcinoma. Mechanistically, when localized to the cytoplasm, LINC00662 has been shown to serve as a ceRNA for gene regulation and to influence RNA metabolism. In parallel, LINC00662 has also been shown to participate in regulating mRNA stability as a mediator of gene expression. Notably, LINC00662 has also been shown to interact with protein and RNA and to participate in a variety of vital signaling pathways, including the MAPK/ERK pathway, the Hippo pathway, and the Wnt/β-catenin signaling pathway.

Although the traditional treatment methods have been greatly developed, at present, the clinical treatment of cancer still faces with many problems. High recurrence rate and low treatment accuracy are still obstacles to further improve the prognosis of cancer patients. Therefore, LINC00662, which is a potential biomarker for early prediction of cancer initiation, is a novel promising target for cancer. However, the studies of LINC00662 are limited and mainly focus on investigating the vital role of LINC00662 in oncogenesis. We consider that the upstream molecular mechanism controlling the expression of LINC00662 remains to be revealed. Moreover, the interaction of ncRNA network and the clinical effectiveness of targeting LINC00662 are also unclear. Though further clinical studies are needed to assess the clinical value of LINC00662 in different tumors, LINC00662 still shows extraordinary promise.

## Author Contributions

YH and WG designed the study. YX and ZS searched the articles and made figures. YH and XY wrote this manuscript. All authors worked collaboratively on the work presented here, read and approved the final manuscript.

## Conflict of Interest

The authors declare that the research was conducted in the absence of any commercial or financial relationships that could be construed as a potential conflict of interest.
